# New insights into bacterial type II polyketide biosynthesis

**DOI:** 10.12688/f1000research.10466.1

**Published:** 2017-02-21

**Authors:** Zhuan Zhang, Hai-Xue Pan, Gong-Li Tang

**Affiliations:** 1 State Key Laboratory of Bio-organic and Natural Products Chemistry, Shanghai Institute of Organic Chemistry, Chinese Academy of Sciences, Shanghai, China

**Keywords:** Type II polyketide biosynthesis, type II polyketide synthases, PSKs, bacterial polyketide biosynthesis

## Abstract

Bacterial aromatic polyketides, exemplified by anthracyclines, angucyclines, tetracyclines, and pentangular polyphenols, are a large family of natural products with diverse structures and biological activities and are usually biosynthesized by type II polyketide synthases (PKSs). Since the starting point of biosynthesis and combinatorial biosynthesis in 1984–1985, there has been a continuous effort to investigate the biosynthetic logic of aromatic polyketides owing to the urgent need of developing promising therapeutic candidates from these compounds. Recently, significant advances in the structural and mechanistic identification of enzymes involved in aromatic polyketide biosynthesis have been made on the basis of novel genetic, biochemical, and chemical technologies. This review highlights the progress in bacterial type II PKSs in the past three years (2013–2016). Moreover, novel compounds discovered or created by genome mining and biosynthetic engineering are also included.

## Introduction

Type II polyketide biosynthesis usually begins by loading an α-carboxylated precursor, normally acetate, onto an acyl carrier protein (ACP), which is subsequently transferred to the active site of a ketosynthase (KS) and then undergoes iterative elongation using malonyl-coenzyme A (CoA) as extender units to afford a nascent poly-β-keto chain ^1– 3^. The following ketoreductases (KRs) and aromatases (AROs), along with oxygenases and in some cases transferases, function together to convert the poly-β-keto chain into the aromatic polyketide core. Finally, post-tailoring modifications composed of redox, group transfer, hydrolysis, and rearrangement reactions endow the polyketide core skeleton with structural diversities that contribute to potent therapeutic properties. Mechanistic studies of polyketide biosynthesis have made great progress in the past decade; however, because of emerging antibiotic and anticancer drug resistance, there is a need to unravel the chemical logic and molecular machinery involved in the biosynthetic pathway; this will form the basis of engineering natural product assembly lines to generate bioactive compounds that could lead to clinical candidates. In the past few years, quite a number of novel catalytic mechanisms and biosynthetic strategies have been discovered, and several biosynthetic pathways have been revealed. Additionally, a growing number of crystal structures of enzymes involved in type II polyketide biosynthesis have been elucidated, which not only contributes a lot to mechanistic characterization but also enables the study of enzymatic evolution at the structural level and provides structural diversities when combined with chemical synthesis.

## Recent advances in enzymes responsible for the formation of aromatic polyketide skeletons

**Figure 1.**
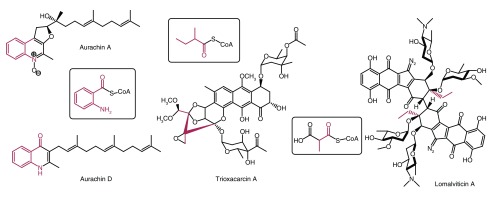
Non-acetate starter units involved in the biosynthesis of aurachins, trioxacarcins, and lomaiviticin A.

An essential strategy to provide structurally diverse natural products is to employ different starter units. Recently, several non-acetate starter units have been identified in type II polyketide biosynthesis. Balskus and Waldman discovered a bifunctional acyltransferase/decarboxylase (LomC) that catalyzes the decarboxylation of methylmalonyl-CoA to generate propionyl-CoA, which then served as a starter unit during lomaiviticin biosynthesis ( Figure 1) ^4^. Tang *et al.* revealed that trioxacarcin biosynthesis may employ 2-methylbutyryl-CoA, a five-carbon precursor, as a starter unit; the ^13^C _6_-L-isoleucine labeling study strongly supported the five-carbon precursor, although there was no direct evidence for the incorporation of 2-methylbutyryl-CoA ( Figure 1) ^5^. Additionally, the crystal structure of AuaEII, the anthranilate-CoA ligase involved in generating anthraniloyl-CoA, which acts as a starter unit during a type II polyketide synthase (PKS) pathway in aurachin biosynthesis, was reported by Tsai’s group ( Figure 1) ^6^.

**Figure 2.**
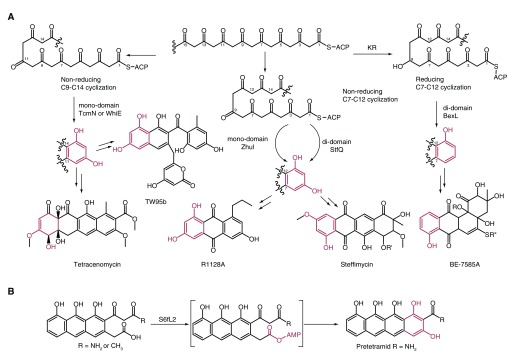
Advances in bacterial aromatic polyketide core biosynthesis. ( **A**) Regiospecific cyclizations catalyzed by aromatases (AROs)/cyclases (CYCs) TcmN, WhiE, ZhuI, StfQ, and BexL. ( **B**) SsfL2 catalyzes the fourth ring cyclization in tetracycline biosynthesis. KR, ketoreductase.

After the formation of the poly-β-keto chain, the AROs/cyclases (CYCs) catalyze the regiospecific cyclization of the nascent polyketide intermediate to yield a defined polyketide product. AROs/CYCs can be separated into C7–C12 and C9–C14 AROs/CYCs based on the first ring cyclization patterns. Otherwise, according to their substrates, AROs/CYCs can be divided into two subclasses: “reducing” AROs/CYCs, which act on one carbonyl-reduced poly-β-keto, and “nonreducing” AROs/CYCs, which act on nonreduced poly-β-keto. Additionally, there are two types of AROs/CYCs – mono-domain and di-domain – on the basis of amino acid sequences. Three non-reducing mono-domain AROs/CYCs – TcmN (C9–C14), ZhuI (C7–C12), and WhiE (C9–C14) – have been reported by Tsai and co-workers in the past few years. Just recently, they presented the crystal structures of two di-domain AROs/CYCs: StfQ (non-reducing C7–C12) and BexL (reducing C7–C12) ^7^. The structure-based mutagenesis and *in vitro* functional assays revealed significant differences between StfQ and BexL. The C-term domain of StfQ functions as a dehydratase/ARO, catalyzing the first cyclization in the non-reducing system; on the contrary, the N-term domain of BexL in the reducing system, instead of the C-term in the non-reducing system, catalyzes the key reaction in the biosynthesis of aromatic polyketides ( Figure 2A). This work greatly enriches our knowledge of the structures and functions of mono- and di-domain AROs/CYCs in bacterial PKSs.

During the biosynthesis of four-ring aromatic polyketides, the fourth ring CYC catalyzes the last ring’s formation to afford the primary polyketide core. Previously, structural and mechanistic studies of the last ring closure in anthracyclines revealed a CYC-mediated base-catalyzed mechanism ^2^. A recent example of an atypical fourth ring cyclization in tetracycline biosynthesis was reported by Tang and co-workers, who demonstrated that SsfL2, conserved in all reported tetracycline biosynthetic gene clusters to date, is an ATP-dependent acyl-CoA ligase family enzyme ^8^. SsfL2 catalyzes an ATP-dependent C1–C18 Claisen condensation to close the fourth ring to form the tetracyclic compound, which serves as the common precursor of tetracyclic natural products. It is proposed that SsfL2 directly adenylates the tricyclic carboxylic acid to facilitate the final carbon–carbon bond formation in the absence of CoA ( Figure 2B). The advanced insight into the logic underlying aromatic polyketide core biosynthesis is therefore an essential factor to engineer the biosynthesis of non-natural analogs of aromatic polyketides.

## Recent progress in tailoring reactions

**Figure 3.**
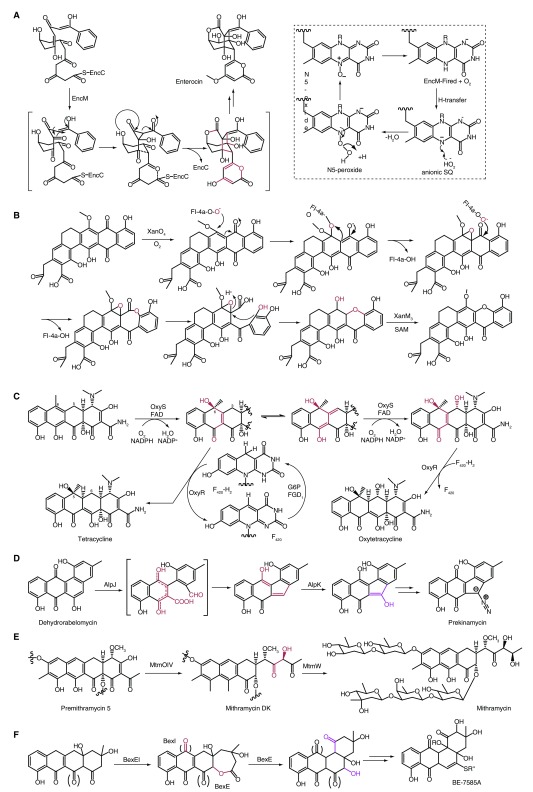
Progress in tailoring reactions catalyzed by flavin enzymes in the type II polyketide synthase system. ( **A**) Oxidative Favorskii-type rearrangement catalyzed by EncM in enterocin biosynthesis. ( **B**) XanO4 catalyzes epoxidation and Baeyer-Villiger (BV) dual reaction during the biosynthesis of xantholipin. ( **C**) Hydroxylation catalyzed by OxyS and reduction mediated by OxyR in the oxytetracycline pathway. ( **D**) B-ring contraction catalyzed by a conserved AIpJ/AIpK pair in kinamycin biosynthesis. ( **E**) MtmOIV catalyzes ring opening in mithramycin biosynthesis. ( **F**) BexE-mediated oxidative reactions during BE-7585A biosynthesis. FAD, flavin adenine dinucleotide; NADP, nicotinamide adenine dinucleotide phosphate; SAM, S-adenosyl methionine.

Post-tailoring enzymes endow the primary aromatic polyketide core with broad structural diversities and biological activities, of which the redox reactions are the most abundant and important processes ^9^. Relentless effort has been made in investigating redox reactions involved in natural product biosynthesis because of its mechanistic and catalytic diversity, and, among them, flavoenzyme-mediated reactions are most often observed ^10^. One extraordinary example is the detailed characterization of EncM, a flavoenzyme catalyzing an oxidative Favorskii-type rearrangement during enterocin biosynthesis. It is proposed that a stable flavin-oxygenating species, the flavin-N5-oxide (Fl _N5[O]_) species instead of the widely believed flavin-C4a peroxide, performs a dual oxidation of the highly reactive poly(β-carbonyl) and initiates a rare Favorskii-type rearrangement ( Figure 3A) ^11^. The catalytic mechanism was demonstrated on the basis of ^18^O _2_ labeling experiments combined with UV-VIS spectroscopy as well as *in vitro* studies using synthetic substrate analogues owing to the high reactivity of the presumed substrate. However, there is no direct evidence for Fl _N5[O]_ species owing to the absence of this flavin modification from the X-ray diffraction data. The further isotope labeling experiments and high-resolution tandem mass spectrometry of proteinase K-digested EncM provide direct evidence for Fl _N5[O]_ species, which may be derived from the direct reaction of O _2_ with the flavin: hydrogen transfer from Fl _red_ to O _2_, resulting in the production of flavin SQ and protonated superoxide, followed by the formation of a flavin-N5-peroxide via radical coupling of the formed protonated superoxide and anionic flavin semiquinone, and the subsequent water elimination affords the Fl _N5[O]_ cofactor ( Figure 3A) ^12^. Recently, another flavin-dependent monooxygenase (MO), XanO4, was demonstrated to catalyze epoxidation and, through Baeyer-Villiger (BV) dual reaction, transform an anthraquinone into a xanthone skeleton during the biosynthesis of xantholipin ^13^. Significantly, homologous comparisons indicated that the XanO4-mediated reaction was supposed to be the general model of constructing a xanthone ring in polycyclic xanthone antibiotic biosynthesis ( Figure 3B). Tang and co-workers reported that the flavin enzyme OxyS and the F _420_-dependent enzyme OxyR are responsible for the final steps in oxytetracycline biosynthesis, demonstrating that OxyS can also catalyze hydroxylation at C5 except C6 and OxyR catalyzes the reduction of C5a–C11a double bond with F _420_ as cofactor; this result broadens the reaction profile of F _420_-dependent enzymes ( Figure 3C) ^14^. Yang and colleagues reported that an AlpJ/AlpK pair conserved in kinamycin-like gene clusters is responsible for benzofluorenone formation during kinamycin biosynthesis ^15, 16^. They demonstrated that AlpJ, a di-domain oxygenase of which the N- and C-terminal halves share high homologies, alone catalyzes B-ring cleavage and contraction. Structural and functional investigations of AlpJ reveal an intramolecular domain–domain interface with a hydrogen bond between His50 and Tyr178 and demonstrate that His50 and Tyr178 are vital for AlpJ activity by site-directed mutagenesis. Otherwise, the p-hydroxybenzoate hydroxylase (PHBH) family flavin enzyme AlpK plays an essential role in preventing spontaneous dimerization and trimerization of the enzymatic product of AlpJ by introducing a C-5 hydroxyl group; another function of AlpK is supplying FADH _2_ through an atypical mechanism relative to the normal flavin adenine dinucleotide/flavin mononucleotide reductase ( Figure 3D). Crystal structures of BVMO MtmOIV with higher resolution and in complex with natural substrate were resolved by Rohr and co-workers ^17^; the substrate-bound structure reveals significant differences to the previous computer model in key residues for substrate recognition and catalysis ( Figure 3E). Tsai *et al.* presented the crystal structure of BexE, which is responsible for the key oxidative rearrangement reaction in forming the angucyclinone framework ^18^ and molecular docking of proposed substrates, and *in vitro* biochemical assays suggest that the linear anthracyclinone 12-deoxy-aklaviketone may act as the possible substrate of BexE; however, the exact substrate of BexE and its mediated mechanism need further exploration ( Figure 3F).

**Figure 4.**
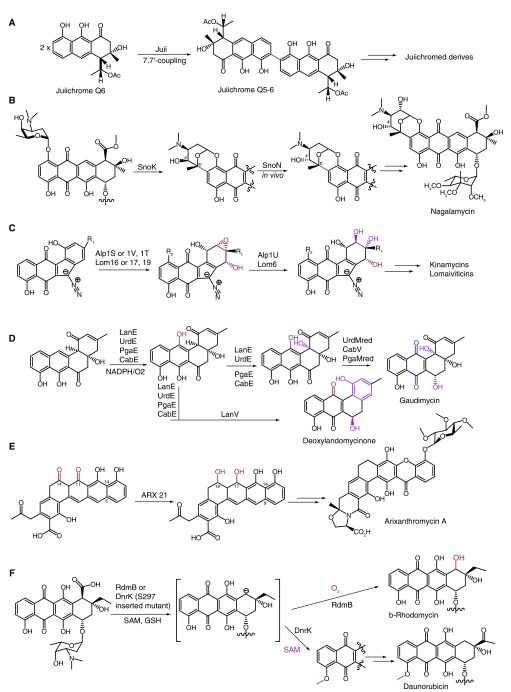
Other enzymes for tailoring reactions in type II polyketide biosynthesis. ( **A**) P450-mediated intermolecular oxidative phenol coupling reaction. ( **B**) Divergent reactions catalyzed by non-heme iron enzymes SnoK and SnoN in the nogalamycin biosynthetic pathway. ( **C**) Epoxy hydrolases catalyzed by AIp1U and Lom6 in kinamycin and lomaiviticin biosynthesis. ( **D**) LanV and its homologous CabV (Urdmred)-catalyzed ketoreductions. ( **E**) ARX21-mediated keto-reductions. ( **F**) Reactions catalyzed by RdmB and DnrK. NADPH, reduced nicotinamide adenine dinucleotide phosphate; SAM, S-adenosyl methionine.

P450 enzymes are widely distributed in secondary metabolism ^19^ but are seldom involved in the biosynthesis of bacterial type II polyketides. Dimerization reactions involved in the biosynthesis of bacterial aromatic polyketides play a critical role in their diverse biological activities; however, the mechanisms conferring these reactions have not been well explored. Müller and colleagues, for the first time, uncovered a P450 enzyme which performs region-specific and stereospecific intermolecular oxidative phenol coupling to generate axially chiral biaryl compounds in *Streptomyces* ( Figure 4A) ^20^. This work expands the repertoire of reactions reported for P450 and provides an illustrative example of dimerization reactions of a bacterial aromatic polyketide. Non-heme iron, α-ketoglutarate (α-KG)-dependent MOs are another large and diverse superfamily of enzymes involved in natural product biosynthesis ^21^, including the type II polyketide tailoring process. Recently, the Fe ^2+^/α-KG-dependent enzyme SnoK was shown to catalyze C5''–C2 carbocyclization in nogalamycin biosynthesis ( Figure 4B) ^22^. In contrast, the homologous SnoN, displaying 38% sequence identity with SnoK, was proved to catalyze an epimerization at C4''. Detailed structural comparison elucidated the catalytic difference deriving from subtle changes in the positioning of the substrates in front of the mononuclear iron ^22^. Another advance in kinamycin and lomaiviticin biosynthesis is the identification of epoxy hydrolases Alp1U and Lom6 ^23^. An α/β-hydrolase family protein, Alp1U, was demonstrated to catalyze the epoxykinamycin to kinamycin F by *in vitro*/ *in vivo* assays, and Lom6 displaying 25% similarity with Alp1U performs the same epoxy hydrolysis reaction in lomaiviticin biosynthesis ( Figure 4C). Structural diversities of aromatic polyketides also derive from reductions, except for oxygenases. Recently, Metsä-Ketelä and co-workers uncovered the molecular logic of the reverse stereochemical outcomes of LanV and its homologous CabV (UrdMred)-catalyzed ketoreductions on the basis of crystal structures of LanV and UrdMred in complex with nicotinamide adenine dinucleotide phosphate (NADP ^+^) and the product mimic rabelomycin ( Figure 4D) ^24^. Chimeragenesis experiments indicate that the opposite stereochemistry configurations mainly result from the conformational changes in the substrates used for the ketoreduction. Very recently, Tsai’s group reported the crystal structure of ARX21, a C-17- and C-19-reducing KR involved in pentangular polyphenol biosynthesis, and demonstrated that ARX21 has a higher substrate specificity compared with the well-known C-9 KR and can recognize the polyphenol scaffold only by *in vitro* experiments ( Figure 4E) ^25^. In a recent report, chimeragenesis experiments were employed to reveal the divergent evolution of an atypical S-adenosyl methionine (SAM)-dependent MO RdmB by Metsä-Ketelä and co-workers ( Figure 4F) ^26^. They showed that an inserted serine S297 mutant of DnrK, a canonical 4-O-methyltransferase from the daunorubicin pathway that exhibits 52% sequence identity relative to RdmB, succeeds in catalyzing the 10-hydroxylation. This result is an illustrative example of divergent enzyme evolution.

**Figure 5.**
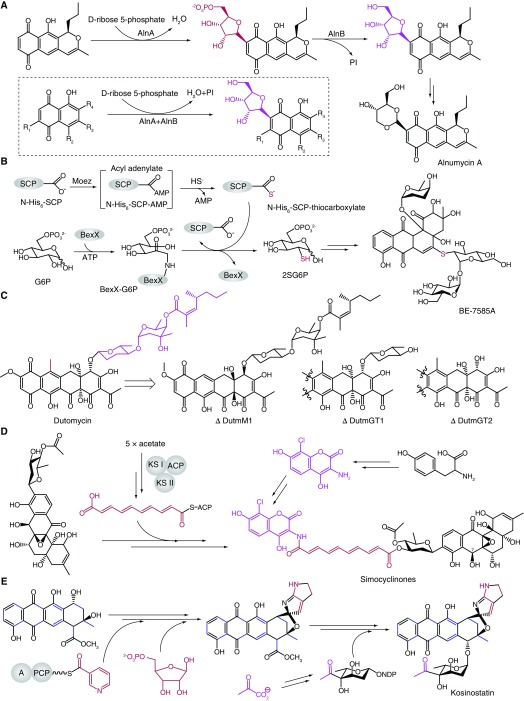
New developments in glycosylation and hybrid pathways for aromatic polyketide biosynthesis. ( **A**) AlnA/AlnB catalyzing C-ribosylation in alnumycin A biosynthesis. ( **B**) 2-Thiosugar biosynthesis in BE-7585A. ( **C**) Methyltransferase and glycosyltransferases in dutomycin biosynthesis. ( **D**) Convergent biosynthesis of simocyclinones. ( **E**) Type II polyketide synthase (PKS)/nonribosomal peptide synthetase (NRPS) hybrid system in kosinostatin biosynthesis. ACP, acyl carrier protein; KS, ketosynthase.

Glycosylation, usually as the last tailoring step, is a general but important process to decorate the natural products, and most natural products derive their activities from sugar substituents ^27^. Recently, an exceptional C-ribosylation in the alnumycin A biosynthetic pathway was illustrated by Ketelä and co-workers ^28, 29^. They demonstrated that AlnA catalyzes the formation of the C8–C1' bond between prealnumycin and D-ribose-5-phosphate via Michael-type 1,4-addition on the basis of the crystal structure of AlnA in complex with D-ribose-5-phosphate. Substrate analogues were also employed to explore the C-ribosylation mechanism ( Figure 5A). However, the exact reaction mechanism has yet to be elucidated. AlnB, a haloacid dehalogenase superfamily protein, performs the following dephosphorylation reaction. Another significant breakthrough in glycosylation is the first complete characterization of thiosugar biosynthesis in BE-7585A by Liu and co-workers ^30^. They demonstrated that the 2-thiosugar is produced by hijacking the sulfur transfer system from primary metabolism. BexX (a putative 2-thioglucose synthase encoded by the BE-7585A gene cluster), the universal activating enzyme MoeZ, and sulfur-carrier proteins from primary metabolism function together with a similar mechanism in thiamine biosynthesis to afford 2-thiosugar ( Figure 5B).

Beyond them, there has been progress in several hybrid pathways of type II polyketides which exhibit complex modifications of a sugar moiety. A dutomycin analogue with improved antibacterial activity was identified from a dutomycin-producing strain, and *in vivo* along with *in vitro* studies also gave new insights into the biosynthesis of dutomycin ( Figure 5C) ^31^. Surprising evolutionary and biosynthetic findings were also made in the hybrid bioactive molecule simocyclinone (an angucyclic polyketide linked with a chlorinated aminocoumarin via a tetraene linker and an acetylated D-olivose sugar). Luzhetskyy and co-workers discovered three new simocyclinones from *Kitasatospora* sp. and *Streptomyces* sp. NRRL B-24484, and the genetic accessibility of the producing strain *Kitasatospora* sp. allows the revelation of the biosynthetic pathway of simocyclinones ( Figure 5D) ^32^. These studies demonstrated that the new stand-alone KSs are responsible for the tetraene chain formation. Just very recently, the detailed structural studies of a C-7 KR (SimC7) with its substrates elucidated new information on the carbonyl reduction ^33^. In addition, biosynthetic studies of kosinostatin, a complex polyketide from marine *Micromonospora* containing a γ-branched octose, revealed a PKS-II/nonribosomal peptide synthetase hybrid pathway using nicotinic acid and ribose as substrates ( Figure 5E) ^34^.

## Recent developments in providing novel aromatic polyketides by biosynthetic engineering, heterologous expression, and genome mining

**Figure 6.**
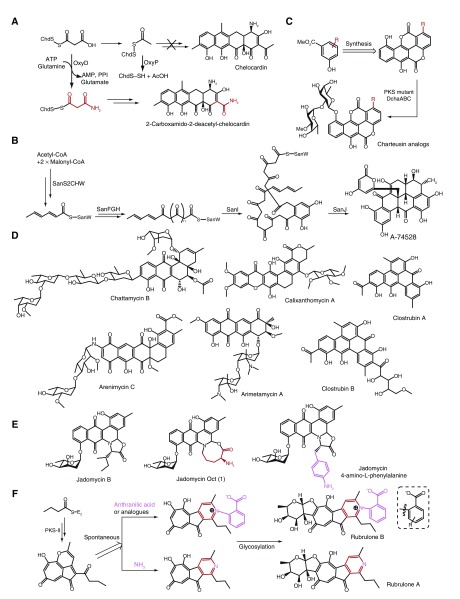
Discovery of novel aromatic polyketides based on biosynthesis. ( **A**) 2-carboxamido-2-deacetyl-chelocardin (CDCHD) provided by biosynthetic engineering. ( **B**) Biosynthetic pathway of A-74528. ( **C**) Chartreusin analogues obtained by synthetic remodeling of the chartreusin pathway. ( **D**) Structures of chattamycin B, arenimycin , arimetamycin A, calixanthomycin A, and clostrubin A and B. ( **E**) Structures of jadomycin B, jadomycin Otc (1), and jadomycin 4-amino-L-phenylalanine. ( **F**) Non-enzymatic pyridine ring formation in rubrolones. PKS, polyketide synthase.

With the development of genome sequencing and elucidations of a large number of natural product biosynthetic pathways, biosynthetic engineering has become a versatile and effective way to either generate non-natural potential drug candidates or optimize the natural product biosynthetic pathway with higher yields along with facilitated operation ^35^. A new “unnatural” natural product, 2-carboxamido-2-deacetyl-chelocardin (CDCHD), with significantly improved antibiotic activities compared with chelocardin, has been generated by introducing an aminotransferase (OxyD) and an acyltransferase (OxyP) from the *Streptomyces rimosus* otc ** gene cluster to a chelocardin-producing strain, where OxyP acts as a thiolase to prevent priming by acetate via the removal of the competing acetyl units, thus only a very low yield of CDCHD can be produced in the absence of OxyP ( Figure 6A) ^36^. Khosla and co-workers identified the minimal set of necessary genes for A-74528 biosynthesis and refactored the minimal A-74528 pathway in the heterologous host *Streptomyces coelicolor* CH999, leading to the production of 3 mg/L A-74528 in the absence of fredericamycin, which accounts for considerably larger quantities than A-74528 in the original strain ( Figure 6B) ^37^. By complementing the chemosynthetic chartreusin and a desmethyl analog of chartreusin with an Δ *chaABC* mutant, Hertweck and co-workers obtained new chartreusin analogues with fine-tuned properties ( Figure 6C) ^38^. This blend of biological and synthetic methods may represent an alternative trend in biological engineering. Charkoudian and colleagues developed an approach to study the evolution of the type II polyketide gene cluster and proposed the predictability of the type II polyketide structure at the genetic level, the limited compatibility of gene combinations, and the key role that structural diversity gene swaps play in evolution ^39^.

Additionally, bioinformatics-guided genome mining and heterologous expression of environmental DNA (eDNA) also contribute to exploring new type II polyketides. By overexpression of a pathway-specific activator gene, chattamycins (members of the angucycline family of antibiotics) were isolated from *Streptomyces chattanoogensis* L10 ( Figure 6D) ^40^. Sequence-guided mining of metagenomic libraries provides a means of recovering specific natural product gene clusters of interest from the environment. Using this strategy, Brady’s group successfully isolated several new pentangular polyphenols (arixanthomycins and calixanthomycins) by heterologous expression of an eDNA-derived gene cluster in *Streptomyces albus* ( Figure 6D) ^41, 42^. Moreover, arimetamycin A, an anthracycline-based natural product, exhibiting improved *in vitro* antiproliferative activity compared to the current clinical anthracyclines, was discovered by heterologous expression of an eDNA-derived *arm* cluster together with genes responsible for deoxy sugar biosynthesis in *S. albus* ( Figure 6D) ^43^. In a recent study, the Hertweck group isolated clostrubins from plant pathogenic anaerobic bacteria ( Figure 6D) and revealed that clostrubins not only enable the survival of anaerobic bacteria under aerobic conditions but also protect the bacteria against other microbial plant pathogens ^44^.

Several non-enzymatic strategies mediating the biosynthesis of type II polyketides have been reported. During jadomycin biosynthesis, the ring cleavage process results in a reactive aldehyde, which couples to a singular amino acid in the culture medium, non-enzymatically forming an imine intermediate that cyclizes into the jadomycin backbone. Using this unique non-enzymatic method and a follow-on amino derivatization, Jakeman and co-workers obtained several eight-membered ring-containing jadomycins and phenylalanine-analogue-derivatized jadomycins ( Figure 6E) ^45, 46^. Very recently, a non-enzymatic pyridine ring formation was found in the biosynthesis of the rubrolone tropolone alkaloids ( Figure 6F) ^47^. Huang and co-workers reported the rubrolone biosynthetic gene cluster and demonstrated that a key biosynthetic intermediate can, in a non-enzymatic fashion, couple to ammonia or anthranilic acid to yield the aglycones of rubrolones. This non-enzymatic construction of an alkaloid pyridine ring provides an alternative strategy for pyridine ring formation in natural products.

## Conclusion

In the future, continuous efforts are still needed to characterize the assembly machinery and catalytic logic of aromatic polyketides at the genetic, structural, and biochemical levels. With the development of gene sequencing technology, combined with optimized heterologous expression systems, eDNA will be an essential source of novel aromatic polyketides. On the other hand, biosynthetic engineering mediated by gene combination from different pathways or activation of the cryptic gene clusters will continue to play an important role in developing new antibiotics with improved activities as well. Other approaches for exploring potent antibiotics also include non-enzymatic strategies and the combination of chemical synthesis and biological transformation.

## Abbreviations

ACP, acyl carrier protein; α-KG, α-ketoglutarate; ARO, aromatase; BV, Baeyer-Villiger; CDCHD, 2-carboxamido-2-deacetyl-chelocardin; CoA, coenzyme A; CYC, cyclase; eDNA, environmental DNA; Fl _N5[O]_, flavin-N5-oxide; KR, ketoreductase; KS, ketosynthase; MO, monooxygenase; NADP, nicotinamide adenine dinucleotide phosphate; PKS, polyketide synthase; SAM, S-adenosyl methionine.
